# Early-Life Stress Paradigm Transiently Alters Maternal Behavior, Dam-Pup Interactions, and Offspring Vocalizations in Mice

**DOI:** 10.3389/fnbeh.2016.00142

**Published:** 2016-07-05

**Authors:** Hanke Heun-Johnson, Pat Levitt

**Affiliations:** ^1^Neuroscience Graduate Program, University of Southern CaliforniaLos Angeles, CA, USA; ^2^Institute for the Developing Mind, Children’s Hospital Los AngelesLos Angeles, CA, USA; ^3^Department of Pediatrics, Keck School of Medicine, University of Southern CaliforniaLos Angeles, CA, USA

**Keywords:** early-life stress, fragmented care, limited bedding, ultrasonic vocalizations, audible vocalizations, maternal care, behavior, dam-pup interactions

## Abstract

Animal models can help elucidate the mechanisms through which early-life stress (ELS) has pathophysiological effects on the developing brain. One model that has been developed for rodents consists of limiting the amount of bedding and nesting material during the first postnatal weeks of pup life. This ELS environment has been shown to induce “abusive” behaviors by rat dams towards pups, while mouse dams have been hypothesized to display “fragmented care”. Here, as part of an ongoing study of gene-environment interactions that impact brain development, we analyzed long observation periods of wild-type C57Bl/6J dams caring for wild-type and *Met* heterozygous pups. *Met* encodes for the MET receptor tyrosine kinase, which is involved in cortical and hippocampal synaptogenesis. Dams with limited resources from postnatal day (P)2 to P9 preserved regular long on-nest periods, and instead increased the number of discrete dam-pup interactions during regular off-nest periods. Immediately after dams entered the nest during off-nest periods in this ELS environment, pups responded to these qualitatively different interactions with an increased number of ultrasonic vocalizations (USV) and audible vocalizations (AV), communication signals that have been associated with aversive and painful stimuli. After returning to control conditions, nest entry behaviors normalized, and dams did not show altered anxiety-like or contextual fear learning behaviors after pup weaning. Furthermore, female mice that had undergone ELS as pups did not show atypical nest entry behaviors in control conditions in adulthood, suggesting that these specific maternal behaviors are not learned during the ELS period. The results suggest that atypical responses of both mother and pups during exposure to this ELS environment likely contribute to long-term negative outcomes in mice, and that these responses more closely resemble the effects of limited bedding on rat dams and pups than was previously suggested. Discerning how different early-life stressors mediate changes in maternal-pup interactions can help elucidate the mechanisms of ELS on brain development and behavior.

## Introduction

Accumulating evidence from both prospective and retrospective clinical studies has demonstrated a significant relation between early adverse experiences, noted as early-life stress (ELS) or toxic stress, and later risk for cognitive, emotional and physical health problems (Shonkoff and Levitt, 2010; Shonkoff, 2012; Moffitt, 2013; McEwen and McEwen, 2015). While long on clinical description, major challenges remain in determining the mechanisms through which ELS has both immediate and long-term pathophysiological effects. These include difficulties in determining the extent to which an individual must be exposed to ELS to elicit long-lasting responses, the influence of genetic factors in modulating the response to ELS, sensitive periods during which exposure has its most powerful impact on developing biological systems, and the factors that influence individual differences in response to ELS.

Animal models provide an opportunity to address biological mechanisms underlying the impact of ELS (Joëls and Baram, 2009; Lyons et al., 2010; Moriceau et al., 2010; Molet et al., 2014). Several models have been developed in rodents to induce ELS in a controlled fashion. Methods include separating pups from their dam for various periods of time during postnatal periods (maternal separation; Levine, 2005; de Kloet et al., 2005), and the introduction of limited nesting materials and bedding during early postnatal periods (Gilles et al., 1996; Ivy et al., 2008; Rice et al., 2008; Schmidt et al., 2011; Molet et al., 2014). Reports document that both maternal separation and limited bedding generate long-term changes in stress response circuitry and downstream biological and behavioral outcomes (Sanchez et al., 2001; Plotsky et al., 2005; Lippmann et al., 2007; Rice et al., 2008; Wang et al., 2011; Loi et al., 2014; Kohl et al., 2015; van der Kooij et al., 2015). Because the reproducibility of obtained rodent behavior can be impacted significantly by human factors (Sorge et al., 2014), models that reduce contact during periods of ELS may have technical, as well as practical advantages.

Limited bedding during early postnatal periods has been a more recent addition to research focusing on ELS. This paradigm, first developed for rats, leads to altered maternal behavior that ultimately correlates with disruption of offspring stress hormone response, brain excitability, and adult behavior (Avishai-Eliner et al., 2001; Cui et al., 2006; Ivy et al., 2008; Dubé et al., 2015; McLaughlin et al., 2015). Two methods to limit access to bedding and nesting materials have been utilized in rat studies: inserting a wire mesh floor in the cage while providing a suboptimal amount of nesting materials (Gilles et al., 1996), or providing only a very small amount of bedding and nesting materials without a wire mesh (Roth and Sullivan, 2005). Measurements of ELS dams in both situations have generally shown a reduction in time spent nursing, licking and grooming the pups by the dams compared to dams in control conditions (Ivy et al., 2008; Moriceau et al., 2009), while additional measurements of ELS rat dams in cages without the wire mesh indicate that dams display “abusive behaviors” toward the pups (e.g., stepping on, and rough handling pups) and spend more time nest building (Roth and Sullivan, 2005; Moriceau et al., 2009; Raineki et al., 2010, 2012). In mice, maternal care is thought to be qualitatively unaltered (in total on-nest time and quality), albeit fragmented, as a result of limited access to nesting materials using a wire mesh floor (Rice et al., 2008; Baram et al., 2012).

In the context of ongoing gene by environment (G × E) experiments to examine the effect of ELS on mice with reduced expression of the MET receptor tyrosine kinase, which mediates synapse development in the forebrain (Judson et al., 2010; Qiu et al., 2014; Eagleson et al., 2016), the present study was designed to understand more fully how limited resources in the home cage of mice alter normal behavior of the dam, and how vocal responses of mouse pups change as a result of these behavioral alterations. Measures of nest entry behaviors were obtained in the home cage, and fear learning and anxiety-like behavior were assessed in the dam to examine potential long-lasting impact on dam behavior that could induce a residual stress response in pups after ending the ELS period. The data suggest that the pups are affected by, and responsive to, atypical physical contact with the dam, and that ELS is induced through atypical contact in this limited resources model in mice.

## Materials and Methods

### Animals

All animal procedures were approved by the Institutional Animal Care and Use Committee at the University of Southern California, and conformed to NIH guidelines. C57Bl/6J mice were housed in a temperature- and humidity-controlled vivarium (20–22°C, 40–60% humidity) that was maintained on a 12-hour light/dark cycle (with lights on at 6 am), in standard ventilated JAG mouse cages (Allentown Inc., NJ, USA) with *ad libitum* access to regular rodent chow and filtered water in drinking bottles. Cages were cleaned weekly, with ample Alpha-Dri bedding (Shepherd Specialty Papers, MI, USA) and one standard pulped cotton fiber nestlet square (Ancare Corp., NY, USA). All experiments were performed using second litters of each dam in the study, which facilitates larger litter size and reduces the variations in maternal care that we have observed with inexperienced, first-time mothers. Breeding was initiated with mice of approximately 8 weeks of age. During breeding, 3–4 females were housed with one male. The males were removed from the cage with females after 10 days, and on approximately embryonic day 16, the females were placed in a clean cage with one nestlet square and housed singly until the pups were born. Pups were weaned on postnatal day (P)21. In the present study, data from one dam and litter were removed from all analyses due to seizures exhibited by the dam.

Data reported here were generated for ongoing G × E studies, focusing on the *Met* receptor tyrosine kinase gene. MET is implicated in synapse maturation in social-emotional forebrain circuitry (Judson et al., 2010; Qiu et al., 2011, 2014) and is a risk gene for autism spectrum disorder (Campbell et al., 2006; Peng et al., 2014). The experimental dams were generated by crossing *Met^fx/fx^* females with *Met^fx/fx^* male mice, resulting in homozygous *Met^fx/fx^* mice, in which exon 16 of the *Met* gene is flanked by loxP sites. These dams do not express Cre, and by all measures (Judson et al., 2009, 2010; Qiu et al., 2011, 2014) are no different than wild-type animals. Experimental *Met^fx/fx^* dams were mated with *Nestin^Cre^* males (generated by crossing *Nestin^Cre^* males with C57Bl/6J females) to produce litters with both control (*Met^fx/+^*) and heterozygous *Nestin^Cre^/Met^fx/+^* (*Met*^+/−^) pups. *Met*^+/−^ pups express approximately 50% of MET protein levels of control brains (Thompson and Levitt, 2015). The proportion of *Met*^+/−^ pups in each culled litter ranged from 1 to 5, out of five pups, with an average of 2.65 *Met*^+/−^ pups. To investigate potential intergenerational effects that have been shown to exist for natural variations in maternal care in rats (Francis et al., 1999), six *Met^fx/fx^* female mice that had undergone ELS as pups were paired with C57BL/6J wild-type males to produce *Met^fx/+^* pups. These second generation pups were reared under control conditions. All mice were genotyped after weaning according to a previously published protocol (Judson et al., 2009) with minor modifications: the final elongation step in the *Nestin^Cre^* reaction was 7 min, and the PCR product was 320 base pairs. For the *Met^fx^* reaction, the duration of the denaturation step during the amplification cycles was set at 1 min.

### Early-Life Stress Paradigm

The ELS procedure was implemented based on Rice et al. (2008). The center floor of the cage was covered with approximately 50 grams of Alpha-Dri bedding to absorb urine, and a stainless steel raised wire floor with 10 × 10 mm square openings and 1 mm wire diameter (Cat# RWF75JMV, Allentown Inc., NJ, USA) was inserted above the bedding. Dams were not able to retrieve the bedding to incorporate in their nests. Two-thirds (1.8 g) of a standard pulped cotton fiber nestlet square was provided in each ELS cage. Control cages had approximately 160 g identical bedding material and a full nestlet square, and both cage set-ups had identical access to food and water. Dams were alternately assigned to control or ELS conditions based on the time of birth of pups. On the morning of the second day (P2) after birth (P0), the experimental dams were weighed and subsequently placed into ELS or control cages. The pups were removed by hand one-by-one from the nest, weighed, and placed onto the wire floor of the ELS cage or on the bedding of the control cage after determining their sex using genital pigmentation intensity (Wolterink-Donselaar et al., 2009). Three male and two female pups were placed in each cage; the remaining pups in the litter were euthanized. The dam and litter were left undisturbed for 7.5 days until the afternoon of P9, when the dam and pups were removed from the ELS cage, weighed, and placed into a control cage with ample Alpha-Dri bedding and one nestlet square. Cage changes were carried out on P16, and pups were weaned on P21. Dams remained in their respective home cages until behavioral testing. Litters from control dams were similarly culled to three males and two females on P2, and received a regular clean cage with one nestlet square on P2, P9, and P16.

### Corticosterone Analysis

Blood was collected from wild-type pups at 8 am on P9, within 5 min after disturbing the cage (control: *n* = 7 pups from five litters; ELS: *n* = 8 pups from five litters). The pups were quickly decapitated with scissors, and trunk blood was collected in an Eppendorf tube with 100 ng heparin (at 2.8 mg/ml in 0.9% NaCl). Samples were centrifuged at 2000 g at 20°C for 10 min, after which plasma was transferred to a clean Eppendorf tube. The plasma samples were stored at −20°C until shipment on dry ice for corticosterone analysis by the Endocrine Technologies Support Core (ETSC) at the Oregon National Primate Research Center/Oregon Health and Science University. Plasma corticosterone concentration was analyzed by ether extraction and radioimmunoassay (RIA). Samples (10–25 μl) were extracted in 5 ml ether in 13 × 100 glass tubes (baked at 500°C for 30 min), dried under forced air, and analyzed by specific corticosterone RIA. Hormonal values were corrected for extraction losses determined by radioactive trace recovery at the same time as sample extraction. Hot recovery was 87.2% and the sensitivity was 5 pg/tube. Intra-assay variation was 5.3%. Because all values were determined in a single assay, there was no inter-assay variation calculated. However, the overall inter-assay variation for the CS RIA in the ETSC is less than 15%.

### Video Recording and Analysis of Dam Behaviors

Experimental dams and respective litters, in their home cage, were moved to a separate, quiet room within the vivarium suite at 11 am, and left undisturbed for 1 h before starting video recording from noon until 4 pm. Water (in a drinking bottle) and food were available *ad libitum* during the recording period. The mice were recorded through the side cage wall on P4, P8 and P12. Several measures were obtained from the video recordings, including the frequency of the dam leaving and entering the nest (defined as the moment both hind paws are leaving or entering the nest, respectively), the total time spent on-nest and off-nest, the duration of each bout, and the duration of the longest uninterrupted on-nest period. An on-nest period was defined as a period that includes at least one on-nest bout of 900 s or longer—but included brief off-nest bouts shorter than 450 s. An off-nest period was defined as at least 450 s of combined off- and on-nest bouts, without any on-nest bouts longer than 900 s. In addition, the duration of short on-nest bouts during an off-nest period were analyzed separately due to the initial observation that many pup vocalizations occurred during this situation; this was termed “on-nest during off-nest period”.

### Nest Quality Score

The nest quality was visually scored through the sidewall of the cage on P2, P4, P9, P12 and P21, at least 48 h after providing dams with new nesting material. We determined the nest quality score for 37 ELS and 37 control litters for an average of 3.00 and 2.89 of the aforementioned time points, respectively. The scoring methodology is based on a protocol by Hess et al. (2008), and ranges from 0 to 5. A score of 0 means that the dam had not manipulated the nestlet square, while a score of 1 indicates that the nestlet square had been manipulated or interacted with, but a specific nest is not evident. A score of 2 is given when the nesting material is present on top of the bedding (or the grid in ELS cages), but no substantial walls are visible (“flattened saucer shape”). The nest quality score is 3 when the walls are just below “half of a hollow sphere”, and 4 when they “reach or are higher than half of a hollow sphere”. A score of 5 is given when the nest resembles a “complete dome” with a small exit opening. In addition, 0.25 points can be added for each quarter of the nest that has a higher score than the base score of the nest.

### Ultrasonic and Audible Vocalization Recording and Analysis

Eighteen litters (nine control and nine ELS litters) were recorded with ultrasonic audio recording equipment and software (UltraSoundGate 116Hb, condenser microphone CM16/CMPA, AviSASLab Pro software, Avisoft bioacoustics (Germany)) to determine the counts and timing of ultrasonic vocalizations (USV) and audible vocalizations (AV) by the pups in relation to the dam’s location. The microphone was suspended slightly above a temporary cage lid in which a hole was present right above the litter (approximately 5”), at the start of the 1-h acclimation period. The video- and audio recordings were synchronized by quickly tapping a pen against the cage in close proximity to the microphone immediately before and after the analysis period. Audio was recorded for the full 4-h duration of the video recording period. One complete on-nest period and one complete off-nest period was analyzed for vocalizations (vocalization analysis time averages 138.2 ± 42.9 min (mean ± standard deviation, with a range of 50.9–226.6 min). The USV and AV were scored by visually analyzing the audio spectrogram and generating a time stamp of the vocalizations (using Ocenaudio and Praat software) that corresponds with the time stamp of video analysis of the dam’s nest entries and location. For this study, an AV (squeal) was defined as a broadband sound formed by regularly spaced harmonics covering the larger part of the audio frequency spectrum of 0–120 kHz, including the human audible range of 0–20 kHz. USV were defined as any type of narrowband call at a frequency in the ultrasonic range (>20 kHz). When one or more pups were temporarily outside of the nest, the vocalizations from the whole litter for the duration of this event were excluded since additional separation calls generated by pups could not be distinguished from calls from the rest of the litter. Pups were outside the nest for 2.0 ± 1.5% of vocalization analysis time for ELS litters, while none of the control pups were outside the nest in the litters that were included in the vocalization analysis (*p* < 0.01). The average number of vocalizations was determined for the total time that a dam spent in each location (on-nest, off-nest, or on-nest during an off-nest period). In addition, the number of vocalizations in relation to the dam’s entry in or exit from the nest was analyzed; these numbers were presented as the average number of vocalizations per nest entry or exit. One outlier was removed from the control group (in the −20 to −10 s time bin) in the temporal analysis of USV in relation to the dam’s nest entry, using Grubbs outlier detection (α = 0.05). The average USV values per nest entry for this time bin for all control dams are: 0, 0, 0, 1.22, 0, 12.33*, 0.09, 0, 0, with the outlier indicated with an asterisk. To successfully perform a repeated measures two-way ANOVA, all time points from this specific litter were removed from the USV vs. entry time point analysis only. Pup genotype (proportion of *Met*^+/−^ compared to wild-type pups in a litter) did not affect the average number of USV or AV per minute in either control or ELS conditions (Supplementary Figure 1).

### Behavioral Tests of Dams

Following pup weaning, each dam was housed singly for 3 days while being acclimated daily to handling in preparation for elevated-plus maze testing on the fourth day after weaning. After the elevated-plus maze test, the dams were housed in sets of three by experimental group (control or ELS environment) until the contextual fear conditioning test, which was performed 7 to 15 days after pup weaning. All behavioral tests of the dams were performed during the light cycle in the afternoon, in designated mouse behavioral testing rooms that were within the same vivarium corridor as the rooms in which all mice were housed. Dams received a clean cage with ample bedding and one nestlet square after completing the elevated-plus maze test. The person running behavioral tests was blind to the environment experienced by each dam.

#### Elevated-Plus Maze

The elevated-plus maze apparatus (San Diego Instruments Inc., CA, USA) was located on the floor in the center of a 10 × 8 ft room with white walls and even LED lighting throughout the room. The arms of the elevated-plus maze measured 30 cm long, 6.5 cm wide, with 3 mm elevated edges along the open arms. The floor of the maze and the walls of the closed arms were opaque white and opaque black, respectively. The height of the maze was 40 cm above the floor, and the walls of the closed arms extended 14 cm from the maze surface. The light intensity at the end of the open arm, the center of the maze, and the end of the closed arm was 6–7 lux, 3 lux, and 0–1 lux, respectively.

On the 3 days prior to the elevated-plus maze test, mice were acclimated to human handling and to the opaque glass beaker used for transporting mice from the cage to the maze, for 1 h a day. Acclimation consisted of transporting the home cage to a room adjacent to the behavior room, placing each mouse into the beaker and letting them exit the beaker, as well as letting mice freely explore the beaker with the cage top closed. On testing day, mice were placed in a quiet room (light intensity of 8 lux) adjacent to the behavior testing room for 3 h prior to testing. Between each mouse being tested, the maze was cleaned with 70% isopropyl alcohol, and water, and allowed to dry. Mice were transported to the elevated-plus maze room in a beaker, and the beaker was placed at a 45° downward angle in the center of the maze, facing away from the experimenter and the door. Mice were allowed to exit the beaker spontaneously, and the experimenter subsequently exited the room and closed the door. Beaker exit times averaged 85.7 ± 83.9 s (mean ± standard deviation), and the latency to exit the beaker was not correlated with measures of anxiety-like behavior. The 5-min test session began once the tail base of the mouse entered the center of the maze. Time spent in the open and closed arms and the center as well as the number of entries into the arms was recorded with overhead video cameras (SuperExwave SS-E473, Sony) and analyzed using automated TopScan tracking software (CleverSys Inc., VA, USA). The time spent in one area on the maze was defined as the moment the center- and tail base markers of the mouse crossed the border of the area until both markers crossed the border of an adjacent area.

#### Contextual Fear Conditioning

Contextual fear conditioning tests were performed over 2 days. One hour before testing, the dams were transported to a room (light intensity of 200 lux) adjacent to the fear conditioning room (400 lux). During the test, freezing behavior was recorded using near-infrared light and cameras (#NIR-100 infrared light source, Med Associates Inc., VT, USA; #SCA640-71fm camera, Basler, Germany) in double-enclosed fear conditioning chambers 24 × 30 × 22 cm in size, with a metal grid floor, and plexiglass and aluminum walls (#ENV065FPU-M, Med Associates Inc., VT, USA). On day 1, mice were acclimated to the fear conditioning chamber for 3 min, after which 5 × 2-s foot shocks of 0.5 mA were administered to the mice via the metal grid floor (#ENV-414S stimulator/scrambler, Med Associates Inc., VT, USA). Freezing duration was recorded during the 3 min acclimation period (“baseline”), and during four 220-s inter-trial intervals and one 220-s post-shock period (average of five periods reported as “training”). Between tests, the chambers were cleaned with 70% isopropyl alcohol, followed by water. After training, dams were returned to their respective home cages for 24 h. On day 2, the dams were placed into the same fear conditioning chambers for 8 min while freezing behavior (“test”) was recorded and analyzed. Freezing behavior analysis on both days was performed in real-time using Video Freeze software (Med Associates, Inc., VT, USA), with a linear method of observation, a motion threshold of 18, and a minimum freezing duration of 30 frames (1 s).

### Statistical Analyses

All data were analyzed using GraphPad Prism 6 (Graphpad Software, Inc.). The average number of dam nest entries per hour, the total duration of the dam’s presence on the nest, and the nest quality differences were analyzed using a two-way ANOVA (environment × postnatal age of pup). A two-way repeated measures ANOVA was used to analyze fear conditioning data (environment × baseline/training/test), USV and AV counts (environment × location of dam), time spent by dam in location category during vocalization analysis (environment × location of dam), the timing of USV and AV in relation to timing of the dam’s entry (environment × time point relative to dam’s nest entry/exit), and the frequency of on- and off-nest bouts of different durations (environment × bout duration, for on- and off-nest bouts separately). A Pearson correlation analysis was performed to analyze the correlation between the number of *Met^+/−^* pups in a litter and the number of USV and AV on P4. The duration of complete on-nest bouts, as well as EPM measures (percentage time spent in arm and number of arm entries) were analyzed using an unpaired Student’s *t-test* (ELS environment vs. control). Corticosterone measures in the control and ELS group were compared using a one-tailed Welch’s unequal variances *t*-test. Difference in duration of ELS and control pups being outside the nest was analyzed using a Mann-Whitney test. The number of nest entries on P4 of next-generation females was compared to control and ELS dams using a one-way ANOVA. Bonferroni *post hoc* analyses were performed on ANOVA tests with significant main or interaction effects (α = 0.05). Data are presented as mean ± standard error of the mean (SEM).

## Results

### ELS Environment Transiently Alters Dam Nest Entry Behavior and Induces a Stress-Response in the Pups

**Figure 1:**
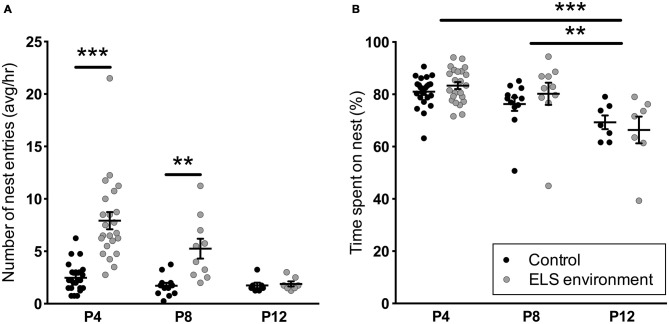
**Early-life stress (ELS) environment induces an increase in the number of nest entries by the dam, but does not affect total on-nest time. (A)** The average number of nest entries per hour between noon and 4 pm (light phase) is increased on P4 and P8 during the ELS period (P2–P9) due to the ELS environment, while the average number of nest entries by ELS dams on P12 are comparable to dams in control conditions. **(B)** ELS environment does not affect the total percentage of on-nest time from noon to 4 pm (light phase) on P4, P8 and P12. The total on-nest time on P12 does, however, decrease in both control and ELS conditions compared to earlier time points. Individual data points represent independent dams, and data are presented as mean ± SEM. P4, *n* = 23/group; P8, *n* = 10–12/group; P12, *n* = 7/group. **p < 0.01, ***p < 0.001.

**Figure 2:**
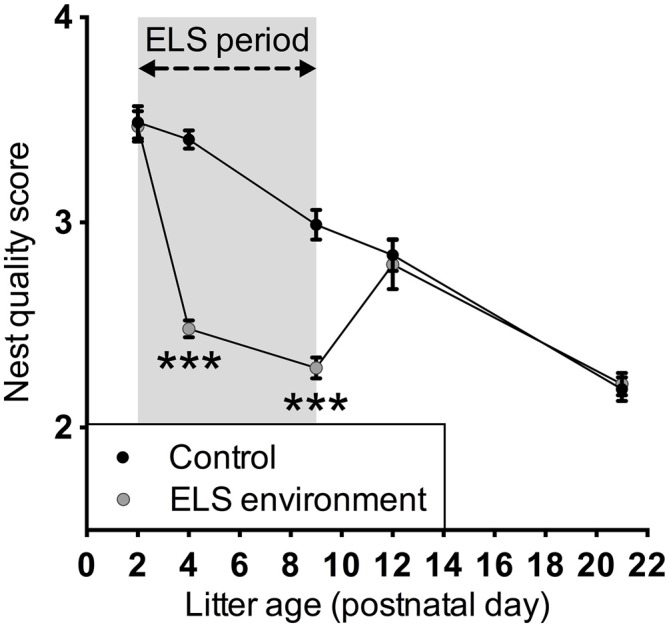
**Limited bedding transiently decreases nest quality during the ELS period.** The ELS conditions only affected nest quality during the ELS period when limited nesting material was available; after the ELS dams return to control conditions, the nest quality was similar to that of control dams. The nest quality was assessed at least 48 h after providing the dams with new nesting materials. Data are presented as mean ± SEM. P2, *n* = 23–24 dams/group; P4, *n* = 28–29 dams/group; P9, *n* = 23–24 dams/group; P12, *n* = 11 dams/group; P21, *n* = 20 dams/group. ***p < 0.001.

Analysis of dam behavior during the 4-h recording sessions confirmed previous reports (Rice et al., 2008) that the ELS environment results in an increase in the number of nest entries by the dam, while the total duration of on-nest time is similar between the control and ELS groups (Figure 1, Table 1 for statistical test details of maternal behavior measurements in the “Results” Section). *Post hoc* tests reveal that on P4 and P8, the number of nest entries are increased as a result of the ELS environment (*p* < 0.001 and *p* < 0.01, respectively), while on P12, 3 days after returning dams and litters to cages with normal bedding, the nest entry frequency of ELS dams returned to control levels (*p* > 0.999). The time spent on the nest on P12 decreased compared to P4 and P8 (*p* < 0.001 and *p* < 0.01, respectively) for dams in ELS as well as in control conditions. The nest quality score was affected by both the ELS environment and age of the litter (Figure 2). *Post hoc* tests indicate that the nest quality score was lower during, and at the end of the ELS period (P4 and P9, respectively) as a result of the ELS environment (*p* < 0.001), but returned to control levels on P12 (*p* > 0.999). At the end of the ELS period on P9, pups in the ELS environment had an increased morning baseline plasma corticosterone concentration compared to control pups (control: 1.55 ± 0.28 ng/ml, *n* = 7; ELS: 3.42 ± 0.96 ng/ml, *n* = 8; *p* < 0.05). These results show that the ELS environment transiently alters dam nest entry behavior and nest quality, and, as has been reported previously (Rice et al., 2008), the altered environment physiologically affects pups by inducing a stress hormone response.

**Table 1:** **Statistical test details of maternal behavior measurements**.

Figure	Measurement	*F* or *t* statistic	*p* value
1A	Number of nest entries	Environment: *F*_1,76_ = 23.65 Litter age: *F*_2,76_ = 10.77 Interaction: *F*_2,76_ = 6.09	*p* < 0.001 *p* < 0.001 *p* < 0.01
1B	Duration of on-nest time	Environment: *F*_1,76_ = 0.28 Litter age: *F*_2,76_ = 15.04 Interaction: *F*_2,76_ = 0.72	n.s *p* < 0.001 n.s
2	Nest quality score	Environment: *F*_1,203_ = 59.38 Litter age: *F*_4,203_ = 106.90 Interaction: *F*_4,203_ = 25.64	*p* < 0.001 *p* < 0.001 *p* < 0.001
–	Pup plasma corticosterone concentration	*t*_8.2_ = 1.88	*p* < 0.05
4A	On-nest bout duration P4	Environment: *F*_1,44_ = 38.25 Bout duration: *F*_9,396_ = 14.41 Interaction: *F*_9,396_ = 10.60	*p* < 0.001 *p* < 0.001 *p* < 0.001
	Off-nest bout duration P4	Environment: *F*_1,44_ = 39.42 Bout duration: *F*_9,396_ = 21.39 Interaction: *F*_9,396_ = 18.09	*p* < 0.001 *p* < 0.001 *p* < 0.001
4B	On-nest bout duration P12	Environment: *F*_1,12_ = 0.09 Bout duration: *F*_9,108_ = 5.28 Interaction: *F*_9,108_ = 2.41	n.s *p* < 0.001 *p* < 0.05
	Off-nest bout duration P12	Environment: *F*_1,12_ = 0.03 Bout duration: *F*_9,108_ = 3.05 Interaction: *F*_9,108_ = 3.05	n.s *p* < 0.001 n.s
5A	EPM open arm time	*t*_18_ = 0.40	n.s
5B	EPM open arm entries	*t*_18_ = 0.84	n.s
	EPM closed arm entries	*t*_18_ = 1.37	n.s
5C	Contextual fear conditioning	Environment: *F*_1,19_ = 3.18 Trial: *F*_2,38_ = 262.8 Interaction: *F*_2,38_ = 0.47	n.s *p* < 0.001 n.s
6A	Number of nest entries - next generation P4	Environment: *F*_2,49_ = 23.88	*p* < 0.001
6B	On-nest bout duration - next generation P4	Environment: *F*_1,27_ = 0.02 Bout duration: *F*_9,234_ = 5.16 Interaction: *F*_9,234_ = 0.51	n.s *p* < 0.001 n.s
	Off-nest bout duration - next generation P4	Environment: *F*_1,27_ = 0.02 Bout duration: *F*_9,234_ = 4.69 Interaction: *F*_9,234_ = 0.89	n.s *p* < 0.001 n.s

### ELS Environment Impacts Dam Nest Entry Behavior During Off-Nest Periods

**Figure 3:**
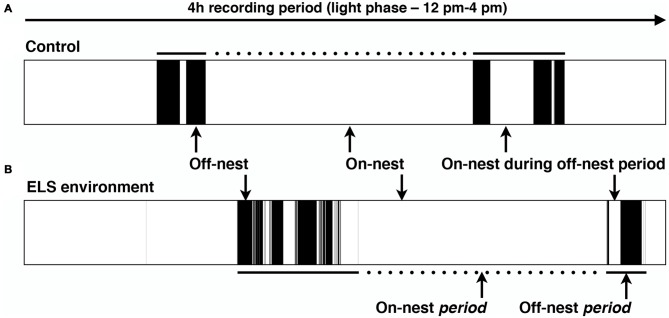
**Representative schematic of the dam’s location in relation to the nest in the home cage.** Black color indicates that the dam is off-nest, while on-nest time is represented in white. Complete on-nest periods (dotted lines) are alternated with off-nest periods (solid lines) by dams in both the control and ELS environment. **(A)** Typical pattern of on/off-nest behavior by a dam in a control and **(B)** ELS environment in a 4-h recording period during the afternoon of the light phase. An ELS environment induces an increase in the number of nest entries by the dam, but only during off-nest periods.

**Figure 4:**
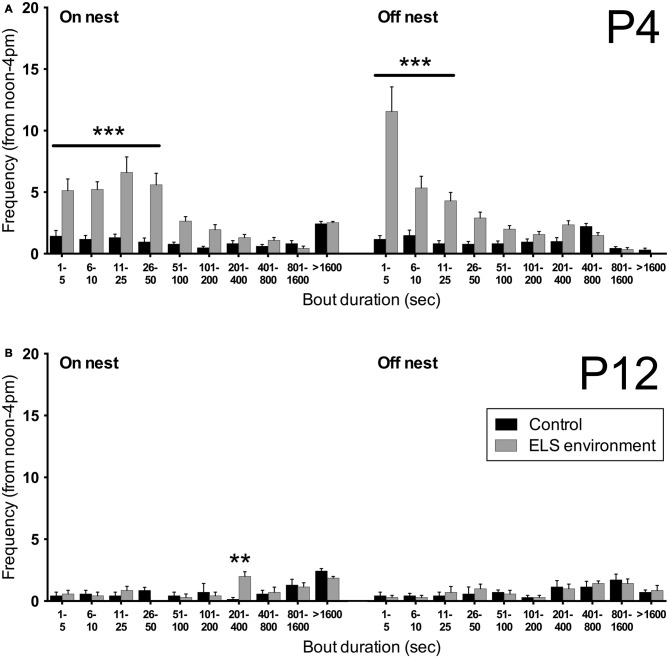
**Limited bedding leads to a transient increase in short on- and off-nest bouts. (A)** The number of short on- and off-nest bouts (≤50 s and ≤25 s in duration, respectively) by the dam is increased as a result of the ELS environment on P4. **(B)** After returning the dams to control conditions on P9, the frequency of on- and off-nest bouts of different durations on P12 are no different between the dams that experienced an ELS environment and control dams, except for a small but significant difference in the frequency of one on-nest duration bin (201–400 s). Data are presented as mean ± SEM. P4, *n* = 23 dams/group; P12, *n* = 7 dams/group. **p < 0.01, ***p < 0.001.

The extended video recording time allowed us to examine in more detail the temporal changes in the patterns of nest entry behavior by each dam. As has been shown previously in rats (Kobayashi et al., 1997), we observed that in control conditions, dams alternated longer on-nest periods with shorter off-nest periods (see representative pattern in Figure 3A). During long on-nest periods, dams slept or cared for the pups by nursing, grooming, and licking them. During shorter off-nest periods, dams spent most of the time eating and drinking, while leaving the litters relatively undisturbed. Self-grooming and nest building occurred both during on- and off-nest periods. Visualization of the nest-entry patterns showed that dams in an ELS environment exited and re-entered the nest more frequently than control dams, but only during off-nest periods (see representative pattern in Figure 3B). The ELS dams appeared to exhibit more careless behavior regarding the pups, often trampling them while traversing the nest during off-nest periods. We also observed frequent disruptive behaviors where the dams, without regarding the pups’ presence, were digging in the nest and thereby displacing pups. These behaviors were only apparent when ELS dams briefly returned to the nest during off-nest periods. Further analyses showed that, despite the increased overall number of nest entries by dams in an ELS environment, the average duration of long, uninterrupted on-nest periods was statistically similar between control and ELS dams (Supplementary Figure 2, *p* = 0.09, Supplementary Table 1). The behaviors towards the pups while nursing and grooming the pups appeared similar between control and ELS dams during on-nest periods. Additionally, on-nest bouts longer than 50 s occurred at a similar frequency during the 4-h recording time in control and ELS dams; only short on-nest bouts that are ≤50 s were observed more frequently in the ELS group compared to the control group (Figure 4A, *p* < 0.001). Similarly, only the frequency of off-nest bouts ≤25 s was significantly increased as a result of the ELS environment (Figure 4A, *p* < 0.001). On P12, 3 days after the ELS period, the frequency of shorter on- and off-nest bouts was largely the same between dams in ELS and control conditions, except for a small but significant difference in the frequency of one on-nest duration bin (Figure 4B, *p* < 0.01 for on-nest bout duration bin of 200–400 s. These analyses of maternal behavior during the post-ELS period show that the altered nest entry behavior of the dam is a transient effect due to the ELS environment.

### Dams Experiencing ELS Environment Exhibit Normal Behaviors after Pup Weaning

**Figure 5:**
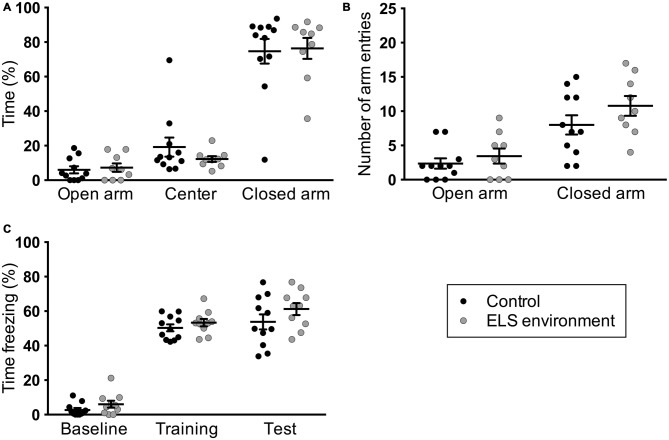
**Dams in limited bedding conditions from P2–P9 do not exhibit altered anxiety-like and fear learning behaviors after pup weaning.** ELS environment does not result in changes in **(A)** the time the dams spent in the open arms of the elevated-plus maze or **(B)** in the number of arm entries. **(C)** Contextual fear learning (“training”) or memory (“test”) was not affected as a result of limited bedding, as measured here by the amount of freezing in a specific context that is associated with foot shocks. Individual data points represent independent dams, and data are presented as mean ± SEM. *n* = 9–11 per group.

The focus in animal ELS models has been predominantly on determining short- and long-term effects on the offspring. To our knowledge, mouse dam behavior after being in an ELS environment with limited nesting materials has not been reported in the literature. To investigate whether the ELS environment impacts dam behavior following pup weaning on P21, we evaluated anxiety-like and contextual fear learning behaviors of the dams. We did not observe a difference in the percentage of time spent in the open arm (*p* = 0.695) and open arm entries (*p* = 0.413) between control dams and dams in the ELS environment (Figure 5A). Additionally, the number of closed arm entries was unchanged due to the ELS environment (Figure 5B, *p* = 0.187), suggesting that there was no difference in overall activity levels between control and ELS dams during this test.

We next evaluated fear learning and memory in the same dams using a contextual fear conditioning paradigm. We did not find a significant main effect of ELS environment on baseline contextual fear, fear acquisition, or fear memory (Figure 5C). The behavioral analyses indicate that dams do not exhibit changes in anxiety-like and contextual fear learning behavior as a result of the ELS environment while raising their pups.

### Mothers That had Undergone ELS as Pups Exhibit Normal Nest Entry Behavior

**Figure 6:**
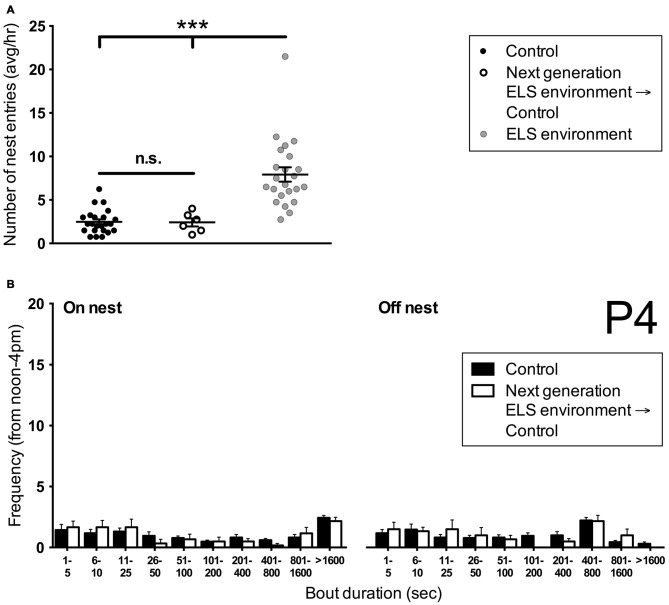
**Dams that have undergone ELS as pups do not show altered nest entry behavior in adulthood when rearing pups. (A)** The average number of nest entries per hour during the afternoon on P4 in females that have undergone ELS as pups is similar to controls. Both control dams and next generation dams enter the nest less frequently than dams in ELS conditions. Note that control and ELS environment data are the same as in Figure 1. Individual data points represent individual dams. **(B)** A more detailed analysis of the frequency of on- and off-nest bouts of different duration shows that next generation dams do not differ in their nest entry behavior from control dams. Control data are the same as in Figure 4A. Data are presented as mean ± SEM. Control and ELS environment, *n* = 23; Next generation, *n* = 6. ***p < 0.001.

To investigate whether ELS has intergenerational effects on nest entry behavior, we analyzed maternal behavior of female offspring that had pups and were subsequently housed in control conditions. The number of nest entries on P4 of these dams was comparable to control (*p* > 0.999) but different from ELS dams (*p* < 0.001) of the previous generation (Figure 6A). In addition, no differences were measured in the number of on- and off-nest bouts of different duration between control dams and dams that had experienced ELS as pups (Figure 6B). These results indicate that nest entry and exit behaviors are not affected by early pup experiences during P2–P9 as a result of the ELS environment (i.e., they are not learned from the dam during this time period).

### ELS Pups Emit More Ultrasonic and Audible Vocalizations

**Figure 7:**
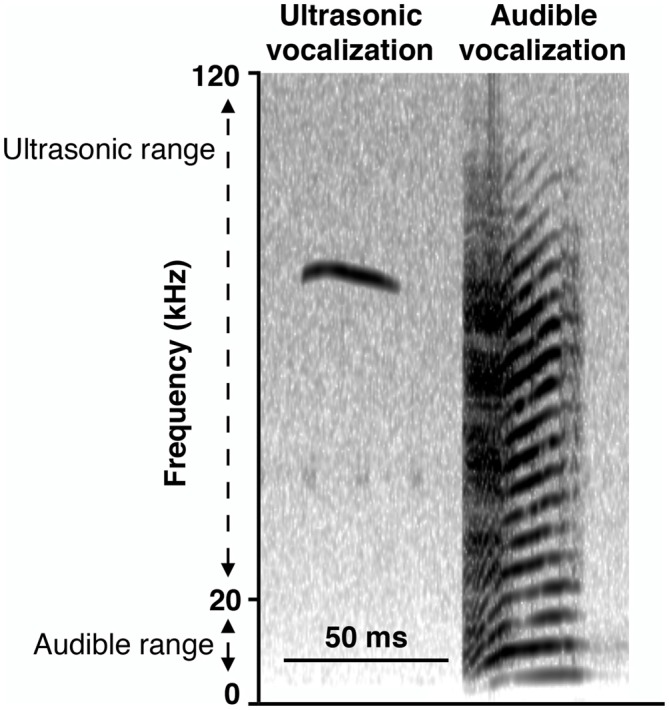
**Representative spectrograms of recording of vocalizations.** Ultrasonic vocalizations (USV; narrowband vocalizations in the ultrasonic range of 20–120 kHz) and audible vocalizations (AV; “squeals” with harmonics covering the larger part of the 0–120 kHz frequency spectrum, including the human audible range of 0–20 kHz) were observed regardless of when the dam was present or absent from the nest, in both control and ELS conditions.

**Figure 8:**
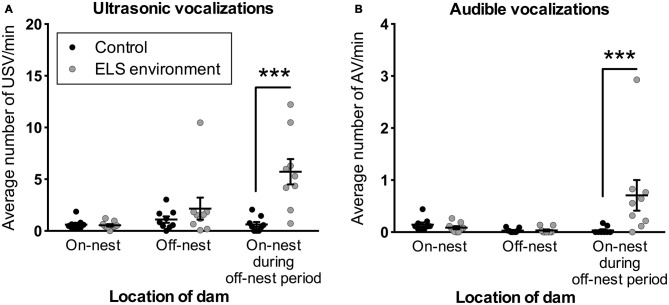
**ELS pups emit more vocalizations when the dam is present on the nest during off-nest periods.** Vocalizations are normalized to time spent in each location in relation to the nest (see also Supplementary Figure 3). The number of **(A)** USV and **(B)** AV is increased when the dam is on-nest during off-nest periods. See also Figure 3 for a schematic illustration of the categories related to the location of the dam. Individual data points represent independent litters, and data are presented as mean ± SEM. *n* = 9 litters/group. ***p < 0.001.

To gain insight into the response of the pups to altered dam nest entry behavior due to the ELS environment, we analyzed USV and AV—see Figure 7 for example spectrograms—for one complete off-nest period and one complete on-nest period per video recording session on P4. We observed both USV and AV during on- and off-nest periods in both control and ELS litters. Throughout the on-nest and off-nest periods, we did not observe differences in the number of USV or AV between the control and ELS litters. However, the average number of USV and AV was significantly higher in ELS litters compared to control litters when the dam was on-nest during off-nest periods (Figure 8, Table 2 for statistical test details of vocalization measures in “Results” Section. See also Figure 3 for a schematic illustration of the timing and location of the dam in relation to the nest). “On-nest during off-nest period” is a specific event when dams briefly return to the nest during an off-nest period, and does not include the longer on-nest period when dams are generally nursing and grooming pups. Specifically, *post hoc* analyses revealed: (1) an increase of USV and AV during this period in ELS litters compared to control litters (*p* < 0.001); and (2) an increase of USV and AV compared to regular on-nest and off-nest time in the ELS group (*p* < 0.001 and *p* < 0.01, respectively). In contrast, the number of USV and AV did not change as an effect of the dam’s location in the control group (*p* > 0.999). Due to slight variability in the duration of on- and off-nest periods between mice, the number of vocalization was normalized to the total time spent in each category, providing a measure of average vocalizations per unit time. Note that no significant overall differences were observed between control and dams in ELS conditions in the duration of each of these categories (Supplementary Figure 3, Table 1), and all individual dams spent time in each category.

**Table 2:** **Statistical test details of vocalization measurements**.

Figure	Measurement	*F* statistic	*p* value
8A	Ultrasonic vocalizations	Environment: *F*_1,16_ = 9.54 Dam’s location: *F*_2,32_ = 8.82 Interaction: *F*_2,32_ = 9.59	*p* < 0.01 *p* < 0.001 *p* < 0.001
8B	Audible vocalizations	Environment: *F*_1,16_ = 4.59 Dam’s location: *F*_2,32_ = 4.00 Interaction: *F*_2,32_ = 5.32	*p* < 0.05 *p* < 0.05 *p* < 0.05
9A	Ultrasonic vocalizations and nest entry	Environment: *F*_1,15_ = 5.10 Timing: *F*_12,180_ = 6.17 Interaction: *F*_12,180_ = 4.74	*p* < 0.05 *p* < 0.001 *p* < 0.001
9B	Audible vocalizations and nest entry	Environment: *F*_1,16_ = 13.26 Timing: *F*_12,192_ = 1.97 Interaction: *F*_12,192_ = 1.84	*p* < 0.01 *p* < 0.05 *p* < 0.05
9C	Ultrasonic vocalizations and nest exit	Environment: *F*_1,16_ = 0.25 Timing: *F*_11,176_ = 1.41 Interaction: *F*_11,176_ = 1.32	n.s n.s n.s
9D	Audible vocalizations and nest exit	Environment: *F*_1,16_ = 0.84 Timing: *F*_11,176_ = 1.34 Interaction: *F*_11,176_ = 0.93	n.s n.s n.s

### Vocalizations Occur After Nest Entry by Dam in ELS Environment

**Figure 9:**
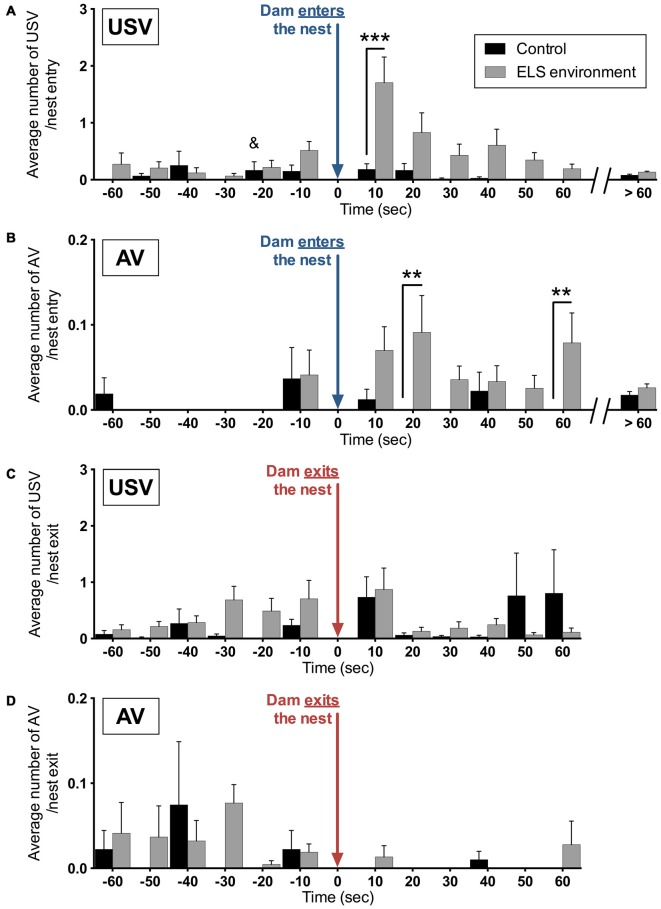
**Vocalizations of ELS pups are increased immediately after dams enter the nest.** The average number per nest entry during the analysis period of **(A)** USV and **(B)** AV are increased at different time periods during the first minute after dams enter the nest as a result of the ELS environment. During the remaining time of the analysis (>60 s after each nest entry), no difference in average vocalizations per entry between the two groups was observed. No significant effect of the ELS environment was observed on the number of **(C)** USV and **(D)** AV before or after dams exit the nest. Data are presented as mean ± SEM and indicates the control group in which one data point in this time bin was determined to be an outlier, which has been removed from this time bin (see “Materials and Methods” Section for more details). *n* = 9 litters per group. **p < 0.01, ***p < 0.001.

To determine whether the dam’s entry in, or exit from the nest induced pup vocalizations, the average number of vocalizations per entry or exit was analyzed in relation to the timing of these nest entries and exits by the dams. In relation to dam entry, the analysis revealed significant main and interaction effects of environment and the temporal relation to the dam’s entry on the expression of USV (Figure 9A). *Post hoc* tests show an increase in the average USV per entry during the first 10 s after the dam enters the nest in the ELS environment (*p* < 0.05). For AV, we also observed significant main and interaction effects of environment and the temporal relation to the dam’s entry (Figure 9B). Specifically, the average number of AV per nest entry is increased between 10–20 s and 50–60 s after the dam enters the nest in the ELS environment (*p* < 0.01 and *p* < 0.05, respectively). In contrast, in relation to the exit of dams from the nest, there was no effect of environment on the average USV or AV per exit (Figure 9C). The increase in pup vocalizations following nest entry by dams in an ELS environment suggests that the manner in which dams enter the nest and initially interact with the pups is qualitatively different from dams in control conditions.

## Discussion

The present study provides a detailed analysis of mouse dam and pup behavior as a result of experiencing a 1-week period with limited access to nesting and bedding material. We show that this ELS environment increases the average number of nest entries of the dam during the off-nest period, while on-nest periods are unaffected. Furthermore, there were no lasting effects on dam non-maternal behaviors that include anxiety-like behavior and fear learning, nor intergenerational effects on nest entry behavior. Finally, in this ELS environment, pups increase their vocalization rate immediately after dams enter the nest during off-nest time, suggesting that atypical physical interactions between the dam and pup contribute to the induction of stress and subsequent long-lasting neurobiological and physiological effects in this ELS paradigm.

### Disruption of Typical Dam-Pup Interaction Patterns Due to ELS Environment

The analyses show that an ELS environment with limited bedding and nesting materials prompts the dam to return repeatedly to the nest during off-nest periods when pups are normally left relatively undisturbed, thereby increasing the number of discrete physical interactions. This ELS environment, however, does not induce apparent changes in interactions during typical uninterrupted on-nest periods. Our results replicate and elaborate on findings by several groups who reported that the frequency of nest exits increased (Rice et al., 2008; Baram et al., 2012; Gunn et al., 2013; Malter Cohen et al., 2013; Naninck et al., 2015; Yang et al., 2015), whereas the total duration of maternal care is not altered during the ELS period (Rice et al., 2008; Gunn et al., 2013; Naninck et al., 2015). In contrast with these studies, as well as with our results, two groups report a decreased (Malter Cohen et al., 2013) and increased (Yang et al., 2015) duration of total on-nest time. We found that 4-h continuous recording sessions, as well as minimal interference with the home cage—including acclimation to the recording room, which allows dams to resume their natural pattern of on/off nest behavior prior to recording—lead to highly reproducible nest entry behaviors. However, by limiting the interference with the home cage, the nest structures, particularly in control cages, obscured viewing discrete dam-pup interactions, other than exiting and entering the nest. This prevented us from analyzing differences in maternal care (e.g., licking and grooming) in more detail.

Under control conditions, dams alternate long on-nest periods with shorter off-nest periods (Kobayashi et al., 1997), and transitions between several maternal and non-maternal behaviors occur in stereotyped strain-specific patterns (Carola et al., 2011). An alternating on/off-nest pattern is necessary to balance the needs for increased energy intake to sustain lactation, nursing pups, and sleeping. This results, during early stages of lactation, in the elimination of increased activity during the dark phase compared to the light phase that is normally observed in mice (Gamo et al., 2013). An increased maternal ventral temperature due to pup presence, as well as the satiety state of the pups, has been implicated as the trigger for rat dams to leave the nest in this stereotypical pattern (Croskerry et al., 1978; Leon et al., 1978; Woodside et al., 1980; Stern and Azzara, 2002; Stern and Keer, 2002). An alternative hypothesis is that the patterns of nest entry and exit patterns by dams are modulated by a motivational switch between maternal behaviors (e.g., nursing pups, licking and grooming, nest building) and consumption of food/water (and other non-maternal behaviors such as activity). Consistent with this, mice have been shown to bar press in order to take care of pups (Hauser and Gandelman, 1985) and to receive food and water, with these behaviors generally being mutually exclusive. It is possible that the grid in this ELS paradigm (or a plastic cage floor often used in rat studies), on which the dam resides is sufficiently aversive for the dam to interrupt the consumption of food and water, or the search for nesting materials, and return to the nest for temporary alleviation.

Regardless of causality, it is clear that the ELS paradigm used in the current study creates an atypical behavioral pattern in the dam that increases discrete, apparently painful, interactions between the dam and pups. This is in contrast to the commonly used maternal separation model, which relies on the *absence* of maternal care to evoke a stress response in the pups. Maternal separation can lead to increased on-nest time (Millstein and Holmes, 2007) and increased licking and grooming of pups immediately after the pups are placed back with the dam (Kosten and Kehoe, 2010), due to an increase in “demanding behaviors” such as suckling, by the pups (Pereira and Ferreira, 2006). It has been shown that this increase in maternal care results in a decrease in pup stress response after repeated daily short-term isolation (15 min), whereas increased maternal care cannot compensate for the effects of daily periods of long-term isolation on the stress response (commonly 3 h or longer). While the maternal separation paradigm is successful in inducing a stress response and leads to enduring behavioral impairments in the pup (Sanchez et al., 2001; Plotsky et al., 2005; Lippmann et al., 2007), the ELS paradigm with limited nesting materials has several advantages; it does not require daily human interaction with the dams and pups, while providing the potential for a continuous stressor to the pups (Gilles et al., 1996; Molet et al., 2014), and it does not affect regular food intake patterns of the pups due to uninterrupted on-nest periods. Additionally, it is important to note that the stressors associated with human neglect and abuse may be more similar to aspects of maternal separation and the ELS paradigm with limited nesting materials, respectively. Future studies would need to be done, however, to explore whether these mouse paradigms can be representative models for different types of stressors in humans.

### Impact of ELS Environment on Pup Vocalizations in Relation to Dam Behavior

Abusive behavior has been referred to as the *de facto* stressor in limited bedding studies using rats (Roth and Sullivan, 2005; Raineki et al., 2010, 2012). Since reports of mouse studies did not reveal any changes in total on-nest time and licking/grooming behaviors (Baram et al., 2012) previously observed in rats (Roth and Sullivan, 2005; Braw et al., 2009; Raineki et al., 2010, 2012; Dalle Molle et al., 2012), it was thought that limited nesting materials induces stress in mice through different means, namely fragmented maternal care. Our results, however, indicate that ELS mouse pups are negatively affected by increased nest entries as they vocalize immediately after the dam enters the nest, while the longer on-nest periods are unaffected. Although the pups could be conveying positive affect in response to the return of the dam, we conclude that this is not likely, since, per nest entry, control pups do not vocalize more immediately after the dam returns. Increased vocalizations have been reported in the limited bedding model in rats (Roth and Sullivan, 2005; Raineki et al., 2010, 2012), although it was not clear from these studies when these vocalizations occur in relation to the dam’s nest entry. The present data demonstrate this temporal relation in mice.

Mouse USV accompany salient behaviors such as affiliative, aggressive, and courtship behaviors (Portfors and Perkel, 2014; Sirotin et al., 2014), although the meaning of specific vocalization patterns is not clear. Several groups have investigated the saliency of USV fragments (Holfoth et al., 2014), the ability of mice to discriminate spectrotemporal patterns of USV (Neilans et al., 2014), and cortical plasticity depending on relevant USV features for specific environmental conditions (e.g., pup vocalizations for lactating dams; Shepard et al., 2015). Rodent pups vocalize when they fall out of the nest, which induces pup retrieval by the dam (Noirot, 1972; Ehret and Haack, 1982; Ehret, 2005; Branchi et al., 2006; Okabe et al., 2013). In addition, male odors, tactile stimuli and low temperature can induce USV in mouse pups (Branchi et al., 1998). Pup vocalizations may also act as inhibitory signals to alert the dam, as female rats that are deafened or have pups that are being prevented from making calls are more likely to injure their offspring (White et al., 1992; Stern, 1997). Dams interrupt licking behavior when pups vocalize, and frequently switch to other behaviors such as nest building (Noirot, 1966). Increased numbers of AV and USV have also been reported when mouse pups were exposed to a variety of painful stimuli (Haack et al., 1983; Han et al., 2005; Williams et al., 2008; Kurejova et al., 2010; Delwig et al., 2012; Tsuzuki et al., 2012). Many of these studies used one or a limited number of ultrasonic frequency recording channels, which does not allow for discrimination between narrowband USV at the selected frequency range, and broadband AV that include (and extends beyond) the same specific frequency range. However, several studies specifically mention the occurrence of both AV and USV as a response to painful stimuli (Haack et al., 1983; Han et al., 2005; Williams et al., 2008; Delwig et al., 2012), and moreover, Delwig et al. (2012) attribute an increased number of pup USV as response to aversive stimuli (e.g., bright light) while AV additionally occur when the stimulus becomes painful (e.g., tail pinch).

The current studies cannot formally exclude that a fraction of USV and AV are generated by the dam, which may be affected by the physical properties of the metal grid. We believe this is unlikely, however, because we would have observed an increase of USV and/or AV specifically when the dam was outside of the nest, on the grid in limited bedding conditions. It also is possible that limited nesting and bedding materials (i.e., a reduced nest quality) alters vocalization rates by changing pup body temperature (Branchi et al., 1998), but there is no difference in the number of USV or AV when the dam is off-nest and the pups are most exposed to the environment.

### Transient Effects of ELS Environment on Dam Behavior

After returning the dam and litter to control conditions on P9, nest entry behavior exhibited by the dams that experienced the ELS environment was comparable to control dams. This is consistent with other findings of a lack of impact due to (prenatal) limited nesting materials (Ivy et al., 2008; Bolton et al., 2013). Furthermore, the ELS environment does not appear to impact long-term non-maternal behavior of the dams, as our data show that anxiety-like and fear learning behaviors in dams were normal after weaning their pups. These results, and the observation that nest-entry frequency by the dam is normalized at P12, indicate that stressors experienced by the pups are only present during, and not after ending the limited bedding conditions due to residual effects of this paradigm on the dam’s behavior. This knowledge will help future studies with delineating the stress exposure when analyzing the effects of stressors on specific stages of brain development. Ivy et al. (2008) have shown a similar lack of effect on anxiety-like behavior in rat dams as measured on the elevated-plus maze, although they did observe a decrease in center time in the open-field test, as well as altered physiological stress markers. Other studies using prolonged maternal separation in rats show no change in the dams in anxiety-like behavior (Eklund et al., 2009; Aguggia et al., 2013), exploration, risk assessment, risk taking or shelter seeking (Daoura et al., 2010). However, additional behavioral tests would need to be done to assess maternal and other general behaviors to rule out lasting effects of limited nesting materials on the dam.

Furthermore, we found that female offspring that underwent ELS as pups did not exhibit altered nest entry behavior in adulthood. This suggests that the stereotypical nest entry pattern that we observed in this study is not learned from the dam during the first postnatal week, but rather is an expression of innate behavior that is transiently altered as a result of different environmental conditions (e.g., the amount of bedding and nesting material). It would be interesting, however, in light of our results that maternal behavior quickly returns to normal after returning to control conditions on P9, to test whether an extension of the ELS time window until P21 (weaning) would negatively impact intergenerational nest entry behavior.

In summary, an environment of limited bedding and nesting materials is an effective early-life stressor that consistently evokes vocal responses in mouse pups that have been associated with aversive and painful stimuli. It appears that the effects of this ELS environment on mouse dams and pups more closely resemble the effects of limited bedding on rat dams and pups than was previously suggested. Our results confirm and extend previous findings that the mouse ELS paradigm used in the present study alternates normal maternal care (during long on-nest periods) and “abusive” behaviors by the dam (through an increased number of physical and/or painful interactions between the dam and pups) with minimal human interference. ELS can be evoked by many different mechanisms in both rodents and humans, and detailed analyses of animal models can help effectively model the impact of stressors on brain development in humans.

## Author Contributions

HH-J and PL contributed to the conception and design of the studies. HH-J performed the experiments and collected and analyzed the data. HH-J and PL interpreted the data. HH-J and PL contributed to the drafting of the work and necessary revision. HH-J and PL did final approval of the manuscript and both are agreeable to be accountable for the studies reported here.

## Funding

The studies were supported by the National Institutes of Health under Ruth L. Kirschstein National Research Service Award F31MH100779 from the National Institute of Mental Health (HH-J), and the Simm/Mann Family Foundation Chair in Developmental Neurogenetics, WM Keck Chair in Neurogenetics, and a grant from Harvard Center for the Developing Child Research Consortium on Biomarkers of Toxic Stress, funded by The JPB Foundation (PL). The Endocrine Technologies Support Core is supported by NIH Grant P51 ODO11092 awarded to the ONPRC.

## Conflict of Interest Statement

PL is a member of the scientific advisory board for Pediatric Biosciences. The institution and authors did not receive 3rd party payments to perform the research reported in this article.
