# Supplementary material for: Early-Life Stress Paradigm Transiently Alters Maternal Behavior, Dam-Pup Interactions, and Offspring Vocalizations in Mice
Source: Front Behav Neurosci. 2016 Jul 5;10:142. doi: 10.3389/fnbeh.2016.00142 (PMC4932116; doi:10.3389/fnbeh.2016.00142)
Supplement: Supplementary file 2 [file DataSheet_1.pdf]

Supplementary Material

Early-life stress paradigm transiently alters maternal behavior, dam-pup interactions, and offspring vocalizations in mice

Hanke Heun-Johnson[1], Pat Levitt[2]*

[1]University of Southern California, Neuroscience Graduate Program, Los Angeles, CA, USA

[2]Institute for the Developing Mind, Children's Hospital Los Angeles, Keck School of Medicine, University of Southern California, Los Angeles, CA, USA

*** Correspondence:**

Pat Levitt, The Saban Research Institute, Children's Hospital Los Angeles, 4650 Sunset Blvd., mail stop #135, Los Angeles, CA 90027, USA. Email: plevitt@med.usc.edu

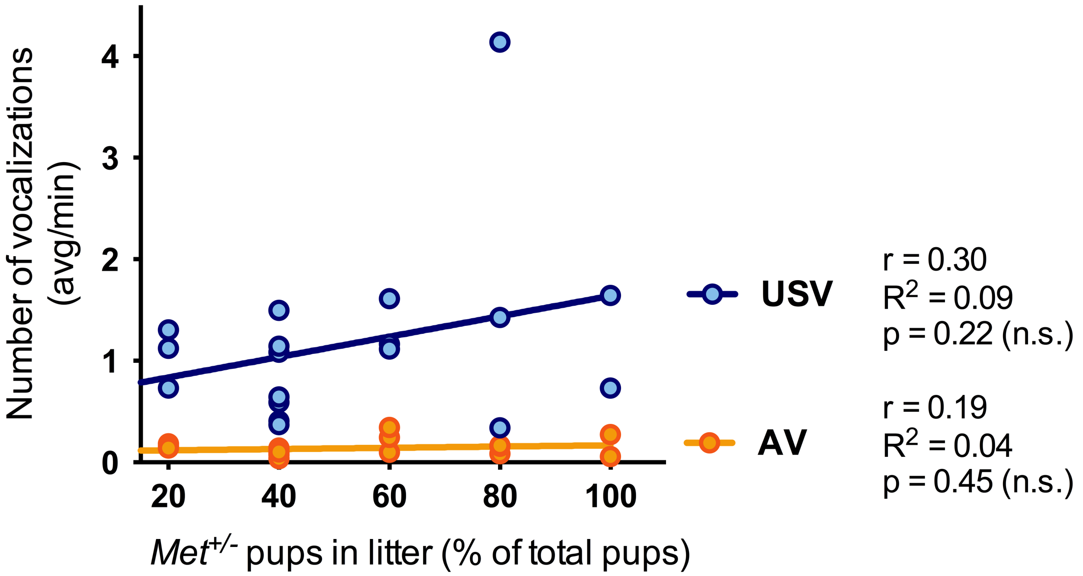

Supplementary Figure 1. Genotype composition of the litter does not affect the number of vocalizations on P4. For ELS and control litters separately, as well as combined (shown here)**,** the average number of USV and AV during the observation period did not correlate with the percentage of *Met*$^{+/-}$ pups in the litter (ELS and control combined: USV: r = 0.30, R^2 = 0.09, p = 0.22 and AV: r = 0.19, R^2 = 0.04, p = 0.45. For ELS and control separately: ELS USV: r = 0.52, R^2 = 0.27, p = 0.15 and ELS AV: r = 0.26, R^2 = 0.07, p = 0.50; Control USV: r = 0.40, R^2 = 0.16, p = 0.28 and Control AV: r = 0.24, R^2 = 0.06, p = 0.53). Litters were culled to three males and two females on P2, blind to genotype. Each individual data point represents one independent litter with mixed genotypes.

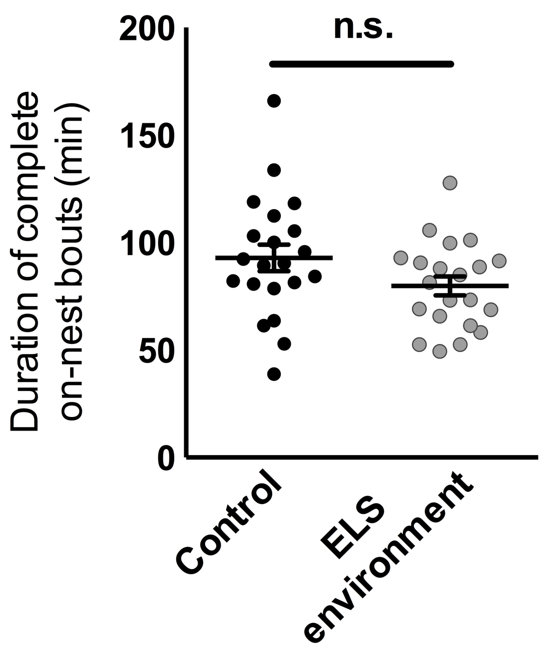

Supplementary Figure 2. The duration of long, uninterrupted on-nest bouts on P4 is similar for dams in a control and ELS environment. For each dam only the longest complete on-nest bout during the four-hour recording window is included in this analysis. Dams that only had long on-nest bouts that already commenced at the start of the recording session, or continued until after the recording session, were excluded. Individual data points represent independent dams, and data are presented as mean ± SEM.

Supplementary Table 1: Statistical test details of supplementary material

Figure	Measurement	F or t statistic	p value
S2	Duration on-nest bouts P4	t_{40} = 1.71	n.s.
S3	Time & location on/off-nest	Environment: $F_{1,16}$ = 0.35<br>Dam's location: $F_{2,32}$ = 148.80<br>Interaction: $F_{2,32}$ = 2.11	n.s.<br>$p < 0.001$<br>n.s.

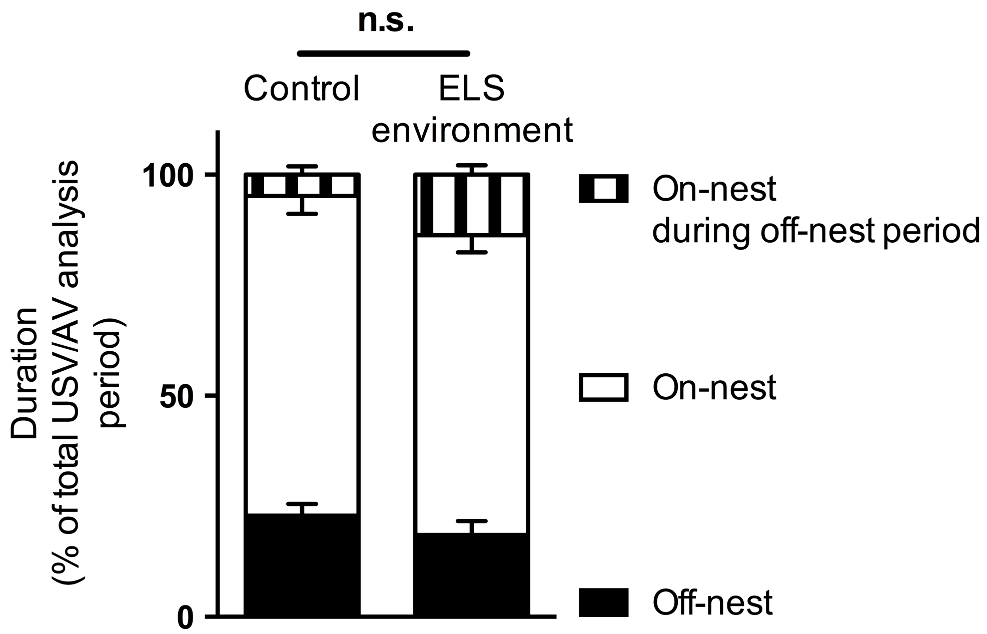

Supplementary Figure 3. The percentage time spent by dams in specific locations (on-nest, off-nest, or on-nest during an off-nest period) during USV/AV analysis varied per dam, but was not significantly different between dams in a control and ELS environment. Data in Figure 8 have been normalized to the absolute time spent by each dam in each category to account for these small variations. Data are presented as mean ± SEM.
